# Impact of a Consumer e-Learning Course on Beliefs, Treatment Choices, and Outcomes Among People With Hip and Knee Osteoarthritis: Qualitative Interview Study

**DOI:** 10.2196/80282

**Published:** 2025-11-05

**Authors:** Rachel K Nelligan, Libby Spiers, Rana S Hinman, Kim L Bennell

**Affiliations:** 1 Centre for Health, Exercise and Sports Medicine, Department of Physiotherapy, School of Health Sciences, The University of Melbourne Parkville Australia

**Keywords:** osteoarthritis, knee, hip, education, qualitative, self-management, e-learning

## Abstract

**Background:**

First-line management for hip and knee osteoarthritis includes lifestyle treatments, such as exercise and weight loss (if appropriate), whereas joint replacement surgery is recommended only for severe symptoms after these options have been exhausted. However, many people with osteoarthritis hold misconceptions about the condition, leading to lower acceptance of nonsurgical treatments, such as exercise, and the mistaken belief that surgery is their only option. Novel patient education approaches that address these misconceptions are recommended to improve uptake of lifestyle treatments, reduce unnecessary surgery, and improve outcomes for people with osteoarthritis. We developed a 4-week self-directed consumer e-learning course on osteoarthritis management. In a randomized controlled trial, using the course led to immediate and sustained improvements in osteoarthritis knowledge. However, participants’ perspectives on the course and an understanding of how it impacted osteoarthritis beliefs, treatment choices, and outcomes were unknown.

**Objective:**

This study aims to explore how an e-learning course for people with hip and knee osteoarthritis may have impacted their osteoarthritis beliefs, treatment choices, and outcomes.

**Methods:**

In this qualitative study, we conducted semistructured individual interviews (N=20) with randomized controlled trial participants with hip or knee osteoarthritis who accessed a 4-week consumer e-learning course on osteoarthritis and its management. Interviews were audio recorded, transcribed verbatim, and thematically analyzed following a framework approach, which was guided by the common sense model of self-regulation.

**Results:**

Four themes were developed from the interviews: (1) participants reshaped their beliefs and attitudes toward osteoarthritis and its management, (2) participants adopted a proactive approach to management, (3) participants developed a more positive mindset, and (4) the course supported learning and shifts in beliefs.

**Conclusions:**

The e-learning course resulted in shifts in participants’ beliefs and attitudes toward osteoarthritis and its management, increasing their confidence in living with osteoarthritis and resulting in a more optimistic outlook on the future. The e-learning course is freely available and could be a useful resource for people with osteoarthritis to enhance their understanding of the condition and its management.

## Introduction

### Background

Osteoarthritis commonly affects the knee and hip joints, causing persistent pain, impaired function, and reduced quality of life [1]. First-line management, recommended for all people with osteoarthritis, includes lifestyle approaches, such as exercise and weight loss (if overweight) [2-5]. Despite joint replacement surgery being recommended only for those with severe symptoms who have exhausted these first-line approaches, many people believe that joint replacement surgery is the only option to manage their symptoms [6,7].

The common sense model of self-regulation has been used to better understand the influence of beliefs about osteoarthritis on the treatment choices and outcomes of people with hip and knee osteoarthritis [6-8]. According to this model, a person’s beliefs about their health condition, including how they label it; their perceptions of its duration, causes, and consequences; and their belief in their ability to control it, can significantly influence treatment decisions and subsequently affect outcomes [9]. For instance, qualitative studies have shown that people with osteoarthritis commonly believe that their condition is caused by “wear and tear,” resulting in “bone on bone.” These misconceptions are associated with lower acceptance of recommended nonsurgical lifestyle treatments, such as exercise, and an inaccurate belief that surgical management is the only viable option [6-8,10]. As a result, novel consumer education methods that address such misconceptions have been identified as key strategies to increase the use of recommended lifestyle treatments and reduce unnecessary surgery [11]. However, the effectiveness of self-directed consumer education interventions in achieving these goals remains unknown; to the best of our knowledge, no qualitative studies have explored the impacts of a digital consumer education intervention for osteoarthritis.

### This Study

To address this gap, our research center developed a 4-week self-directed consumer e-learning course about osteoarthritis and its recommended management [12]. We subsequently evaluated this course in a randomized controlled trial (RCT) of 124 people with hip or knee osteoarthritis and found that using the course led to immediate and sustained improvements in osteoarthritis knowledge when compared to a standard osteoarthritis education intervention, that is, provision of an online osteoarthritis pamphlet available from a reputable consumer organization [13]. Guided by the common sense model, the aim of this study was to gain a richer understanding of the perspectives of people who used the e-learning course within the RCT and explore how it may have impacted their osteoarthritis beliefs, treatment choices, and outcomes.

## Methods

### Ethical Considerations

The institutional human research ethics committee approved this study (responsible committee: STEMM 2; ethics ID 26676). All participants provided informed consent via an online form within REDCap (Research Electronic Data Capture; Vanderbilt University) software hosted at the University of Melbourne [14,15]. Participants received an Aus $50 (US $32.40) gift voucher as a token of appreciation upon completion of their interview.

### Study Design

This study was approached through an interpretivist paradigm, focusing on the subjective perspectives of people within their specific context [16]. It was nested within an RCT that evaluated the effectiveness of a consumer e-learning course on osteoarthritis knowledge and pain self-efficacy in people with hip or knee osteoarthritis; the trial was registered on the Australian New Zealand Clinical Trials Registry (ACTRN12622001490763). Reporting complied with the COREQ (Consolidated Criteria for Reporting Qualitative Research) guidelines [17].

### Participants and Recruitment

Participants in this study were a subsample of those who participated in the RCT, were allocated to the e-learning group, and had completed their involvement in the trial (ie, completed 13-week assessments) within the past 4 weeks. Participants were purposively sampled to ensure variation across gender, age, duration of symptoms, and perceived usefulness of the course (as rated by an outcome measure collected as part of the RCT’s 5-week assessment), with sampling monitored to include participants across the spectrum. The sample size was dictated by theoretical saturation [18]. This was monitored during the coding process by 2 independent researchers, with recruitment ceasing once both agreed there was mounting evidence that new data were providing minimal new insights to address the research question.

Initial recruitment for the RCT was conducted nationwide in Australia via internet sources (eg, social media and online newspapers) and our center’s volunteer database. To be included in the RCT, participants needed to live in Australia and have (1) a native (unreplaced) hip or knee joint meeting osteoarthritis clinical criteria (aged ≥45 years, activity-related pain, and morning joint stiffness ≤30 min) [5], (2) pain in the joint for 3 months or more and on most days in the past month, (3) access to a computer with internet and email, and (4) the ability to provide consent and complete assessments. Participants were excluded from the RCT if they self-reported systemic arthritis (eg, rheumatoid arthritis or gout), were scheduled for lower limb joint surgery in the next 13 weeks, had completed an online education course about osteoarthritis that involved at least 2 hours of learning in the past 12 months, or were unable to easily read and understand English.

### e-Learning Course Intervention

The course is accessible for free on the online education platform FutureLearn (Global University Systems) [19]. During the RCT, the course was available via FutureLearn’s standard tiered access options. This included a free “'limited access” option, where each of the 4 modules was progressively unlocked weekly, and access ceased 4 weeks after registration, with a “buy this course” option, providing immediate unrestricted access. After randomization, participants in the RCT’s e-learning group were sent an email containing the course link, instructions on how to register for the free “limited access” option, and the recommendation to complete module 1 within 7 days, followed by subsequent modules weekly.

The full description of the e-learning course content and its development has been published elsewhere [12]. In summary, its development was informed by behavior and learning theory, osteoarthritis clinical practice guidelines, and consumer consultation. The course includes four modules: (1) learning about osteoarthritis, (2) physical activity and exercise for osteoarthritis, (3) body weight and osteoarthritis, and (4) additional management strategies and conclusion. Content is presented using a variety of formats, including written text, videos, infographics, learning activities (eg, quizzes and polls), and nonmoderated discussion boards. Each module contains a variety of recommended resources, including external links (eg, to evidence-informed self-directed online exercise programs, podcasts, and dietary guidelines), downloadable resources (eg, physical activity or exercise logbooks and action plan templates), and “finding out more” information (eg, information about the range of health care professionals who can provide further help with each topic and references with links to scientific papers that support content). The time required to complete all 4 modules and learning activities is approximately 4 hours (1 h per module). However, this is highly dependent on the user’s level of interaction with the content and recommended resources.

### Data Collection

Participants’ demographic data were extracted from data collected within the RCT. Semistructured, one-on-one telephone interviews were conducted by one researcher (LS) who had no involvement in the development of the e-learning course or its RCT evaluation. Interviews followed an interview guide (Textbox 1), which was developed by members of the research team (RKN, KLB, and RSH) and aimed to explore the potential impact of the e-learning on osteoarthritis beliefs, treatment choices, and outcomes as guided by the common sense model of self-regulation. Interviews were audio recorded and transcribed verbatim by an external transcription service. Deidentified audio files and transcripts were stored in secure password-protected computer files on the university server, and only deidentified data were used for analysis.

**Semistructured interview guide.**

**Introduction**

As part of your involvement in the Osteoarthritis Education study, you were asked to access the online course, Taking Control of your Osteoarthritis. This interview aims to get your feedback on the course and help us understand how the course might have influenced how you think about your knee/hip condition and how to manage it.

Before we start, I’d like to remind you that this interview will be audio recorded and then transcribed. Your responses during this interview will be kept confidential. Any data generated from this interview (e.g., the interview transcript) will not contain your name or any information that could identify you.

At any point, if you would like to skip a question or take breaks during the interview, please let me know. You are also able to cease the interview or withdraw from this study at any point with no adverse effects.

Do you have any questions before we start the interview and I start recording?

**Overview**

1. What were you hoping to gain when you enrolled into the study?

2. Before you joined the study, where had you received most of your information about OA and how to manage your symptoms?

What information did you receive about:

- exercise
- weight management
- surgery?

3. Overall, what did you think of the course you received as part of this study?

- What did you like?
- What didn’t you like?

**Common Sense Model of Self-Regulation domain: OA** **beliefs/illness representation**

4. When you joined the study, what things did you want to learn about OA and its management?

- How well did the course meet your expectations? How/How not?

5. Can you describe if the course provided you with any new information about OA?

Any new information about:

i. what causes it

ii. its symptoms

iii. how it progresses

- Did any information surprise you?

6. Can you describe how the course influenced or changed your thoughts about treatment options for OA?

- Physical activity/Exercise?
- Weight management?
- Medications?
- Surgery—What about your thoughts about needing a Total Joint Replacement in the future?

**Common Sense Model of Self-Regulation domain: Treatments/Coping strategies**

7. Can you describe if the course has changed how you manage or plan to manage your joint symptoms?

- Can you describe if you are doing anything differently?
- Do you plan to do anything differently in the future?

Example prompts: seek health professional support/advice; changes in medication, physical activity, diet?

8. How could the course have better supported you in making choices about how to manage your osteoarthritis?

**Common Sense Model of Self-Regulation domain: Outcomes**

9. What has changed for you because of the information provided in the course? Why?

- Has the information provided in the course lead to any impact on you physically?
- Has the information provided in the course lead to any impact on you emotionally?

**Wrap-up**

10. After completing the Osteoarthritis Education Study, do you have any unanswered questions about OA and its management?

11. Do you have anything else you would like to add about the online course?

### Data Analysis

Participants’ demographic data were summarized using descriptive statistics. Thematic analysis followed a framework approach [20], which has previously been used in qualitative research applying the common sense model in hip or knee osteoarthritis [6,7]. Data were coded by 2 independent researchers with training and experience in qualitative methods (RKN and LS). Data collection and analysis occurred concurrently following the steps mentioned subsequently. First, the 2 independent researchers familiarized themselves with the interview data by reading transcripts and listening to sections of audio recordings, as required. Second, they separately conducted open coding using NVivo software (Lumivero) by rereading the transcript line by line and applying a “code” to summarize passages of data interpreted as important. Third, after the first several transcripts were coded, both researchers met to develop a working analytical framework. This involved the researchers comparing their independently applied codes and agreeing on codes to apply to subsequent transcripts. During this stage, inductively generated codes were also grouped into a priori categories guided by the common sense model: osteoarthritis beliefs, treatment choices, and outcomes. Osteoarthritis beliefs were further divided into 5 subcategories: identity, causes, timeline or progression, consequences or impacts on life, and perceived controllability. Fourth, both researchers continued to independently code subsequent transcripts according to the agreed framework and met two more times to modify the framework to include newly identified codes and discuss whether data saturation had been reached. Fifth, once data collection ceased, one researcher (RKN) organized codes and illustrative quotations into a matrix template within Microsoft Excel, with a priori categories as row headings, codes as row subheadings, and participant identifiers as column headings. This was done to facilitate the identification of patterns within and across participants and categories. The same researcher (RKN) then formulated potential overarching themes, which were reviewed by the interviewer and coder (LS). Finally, the independent coders met with the other members of the research team (KLB and RSH) to discuss the potential themes (with reference to the matrix template), which were refined until all members agreed on the final themes. To minimize bias, reflexivity was maintained by acknowledging the positions of team members and discussing how previous involvement in the development of the e-learning course might influence interpretation.

## Results

### Overview

**Figure 1:**
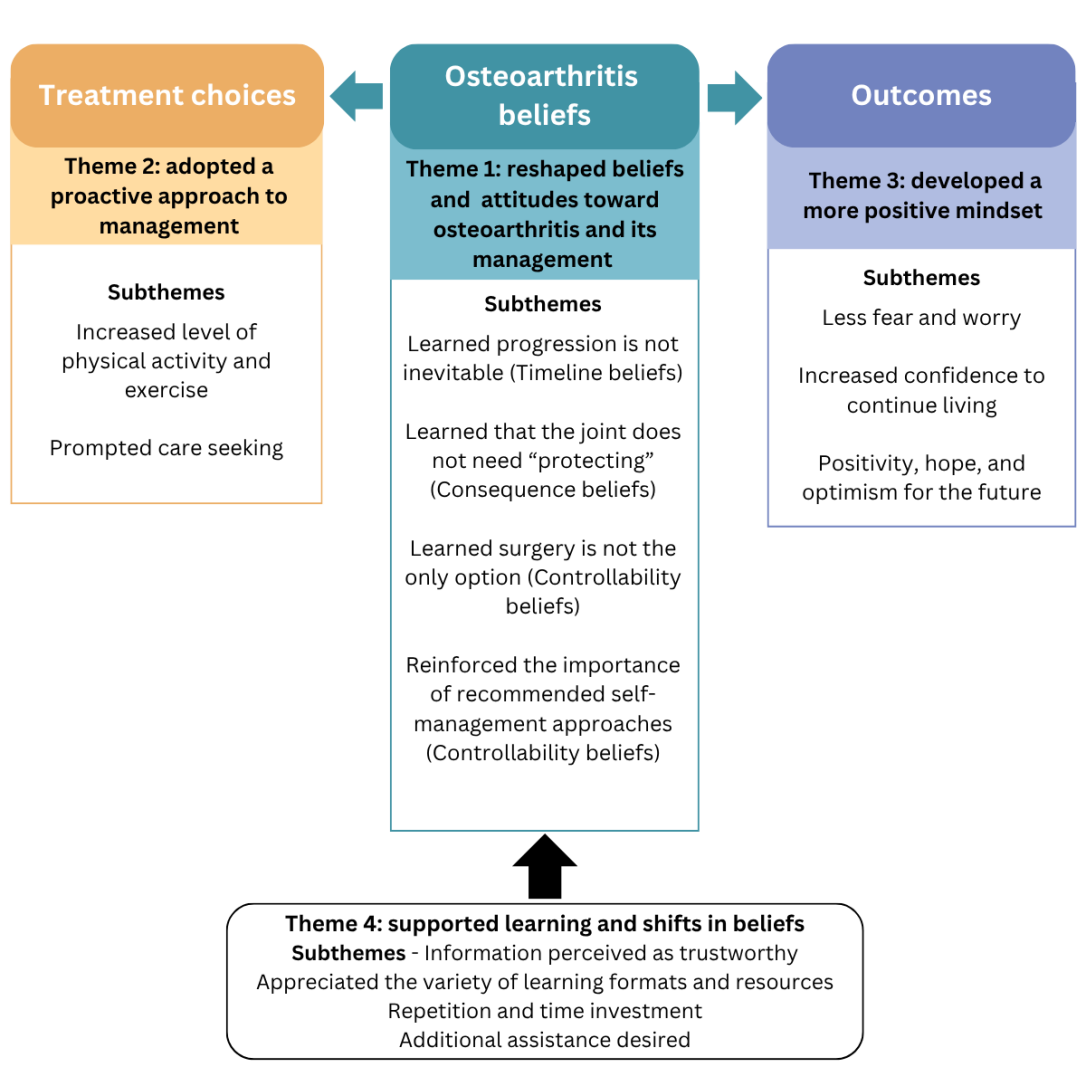
Themes (with subthemes) and their interactions within the common sense model.

A total of 20 participants were interviewed (Multimedia Appendix 1). The mean age of participants was 70 (SD 73) years, 16 (80%) were female, and 14 (70%) lived in metropolitan areas. Four themes were developed with up to 4 subthemes within each. Figure 1 presents these themes and their interactions within the common sense model. Multimedia Appendix 2 provides a summary of themes, subthemes, and exemplary quotes.

### Theme 1: Reshaped Beliefs and Attitudes Toward Osteoarthritis and Its Management

#### Learned Progression Is Not Inevitable

Most participants reflected that before accessing the course, they thought that osteoarthritis was an expected part of aging, and as one gets older, osteoarthritis inevitably worsens. Participants described how the course challenged these beliefs and provided them with a new understanding that osteoarthritis symptoms can be managed, stabilized, or may even improve over time.

#### Learned That the Joint Does Not Need “Protecting”

Participants reflected that, before accessing the course, they had been limiting their physical activities (eg, walking less, stopping running, or reducing work hours) to prevent further joint damage or deterioration to what they believed to be a vulnerable joint. Through the course, they learned that their joint affected by osteoarthritis did not need to be protected in this way.

#### Learned Surgery Is Not the Only Option

For all participants, the course challenged their belief that joint replacement surgery is the sole and inevitable solution—beliefs that were typically based on health professional advice or experience of family or friends undergoing the surgery. Participants felt encouraged and reported a sense of relief in having learned that surgical intervention was not necessarily needed and demonstrated an increased awareness of when surgery may be appropriate.

#### Reinforced the Importance of Recommended Self-Management Approaches

For all participants, the importance of staying active, engaging in regular exercise, and maintaining a healthy weight for joint health was reinforced. Participants described a greater understanding of the mechanisms underlying why exercise and weight management were recommended, which further reinforced the importance of these approaches.

### Theme 2: Adopted a Proactive Approach to Management

#### Increased Level of Physical Activity and Exercise

Participants reported an increased level of exercise and physical activity, which they linked to their changed beliefs (ie, the new understanding that they could be more active without causing damage), and many realized that the activities they wanted to do (but may have stopped) may be beneficial rather than exacerbating their symptoms and damaging their joints. Participants reported increasing their exercise and physical activity levels in a variety of ways, including starting new exercise routines (such as the exercise programs referred to in the course), returning to previous activities that they had ceased (eg, running), adjusting the frequency or intensity of the physical activities they were already doing (eg, spending more time in the garden or walking more), or using movement to alleviate pain (eg, breaking up sedentary time to alleviate joint pain and stiffness). For one participant, adoption of exercise as treatment led to disappointment, as despite their efforts, they did not experience the anticipated improvements that they believed were suggested as possible within the course.

#### Prompted Care Seeking

Many participants discussed that the course motivated them to seek guidance from a physiotherapist for osteoarthritis advice and tailored exercise plans. In addition, 3 participants indicated that the course validated their decision to consult with an orthopedic surgeon to explore joint replacement surgery as the next step in managing their osteoarthritis. They felt reassured in their decision, having tried the nonsurgical approaches recommended in the course without achieving their desired improvements and when the course did not offer further insights into new or alternative treatments.

### Theme 3: Developed a More Positive Mindset

#### Less Fear and Worry

All participants described a reduced sense of worry and fear about having osteoarthritis because of shifted beliefs. Many described a change in how they viewed joint pain or discomfort; they were no longer fearful that it was a definite sign of “damage” and progression. Participants reported increased confidence to push through some discomfort to engage (or re-engage) in various physical activities. Many participants also reflected on the relief they felt at no longer being worried about the inevitability of a surgery.

#### Increased Confidence to Continue Living

Participants described a boost in confidence and feelings of empowerment and no longer feeling restricted by their previous beliefs about osteoarthritis (ie, that the joint needed to be protected and that surgery was inevitable). They described a sense of being granted permission to continue living their lives fully despite having osteoarthritis. All participants described how the course reinforced that they were on the right track and that they could keep doing the things they enjoyed (or start doing them again). This included restarting, continuing, or increasing activities such as walking, jogging, dancing, and camping and a reduced stigma associated with using a walking cane.

#### Positivity, Hope, and Optimism for the Future

Overall, participants described feeling happier and more positive about life, and most described a newfound optimism for the future, no longer viewing osteoarthritis as a “death sentence” and no longer believing that surgery was something that was inevitable.

### Theme 4: Supported Learning and Shifts in Beliefs

#### Information Perceived as Trustworthy

Participants perceived that the course was created by a credible source (ie, researchers at a university), which increased their trust in the information provided.

#### Appreciated the Variety of Learning Formats and Resources

Overall, participants valued the variety of learning materials, such as linked resources, video stories, discussion boards, and quizzes. These options allowed them to tailor their learning experience and “get as much or as little” out of it as suited their needs. However, individual preferences varied; some found the discussion boards beneficial for learning from others’ experiences, while others viewed them as platforms for personal complaints. Similarly, opinions diverged on video content, polls, quizzes, and the links provided to external resources, with some finding them valuable and others preferring not to engage with them.

#### Repetition and Time Investment

Similar information presented across various formats (text, images, and videos) resulted in many participants reporting that they found the course repetitive and time consuming. While some viewed this repetition positively, as a reinforcement of learning, others were frustrated and reported “skimming” various sections. The repetition became particularly bothersome when the content did not align with individual needs. For instance, several participants felt the weight management section was irrelevant to their circumstances, some did not like being asked for feedback “repeatedly” (eg, multiple prompts to engage with discussion boards, polls, and quizzes), and some found the external links hard to navigate.

#### Additional Assistance Desired

Most described that additional support to reinforce, supplement, or personalize course content would have been helpful. However, there were diverse opinions on the preferred form of this support. Suggestions ranged from having the discussion boards moderated to provide personalized responses to posed questions and having initial contact with a health professional to discuss individual concerns to having follow-up contact with a health professional to ask any outstanding questions or to support putting learning into action. Many also desired ongoing access to the content to enable them to revisit it as needed.

## Discussion

### Principal Findings

In this study, we interviewed people with hip or knee osteoarthritis who used an e-learning course about osteoarthritis and its recommended management within an RCT. This study explored the potential impact of the course on participants’ beliefs, treatment choices, and outcomes related to osteoarthritis. Overall, participants valued the course, describing the ways it reshaped their beliefs and attitudes toward osteoarthritis and its management, which resulted in a more positive and optimistic mindset and increased engagement in physical activity. The perceived credibility of the course and the variety of learning formats and resources provided within it facilitated a reshaping of participants’ beliefs. However, the variety of learning resources was also perceived as repetitive and time consuming by most participants.

To the best of our knowledge, this is the first study to qualitatively explore how a self-directed e-learning course for consumers can influence health condition beliefs, treatment choices, and outcomes of osteoarthritis or any other chronic condition. One study has explored, more generally, the perceptions of 20 people newly diagnosed with rheumatoid arthritis toward an e-learning course designed to support self-management [21]. Similar to our findings, the aforementioned qualitative study revealed overall positive participant perceptions toward e-learning. The participants with rheumatoid arthritis trusted the content developed by the program creators (ie, rheumatoid arthritis researchers and health professionals), who were perceived as credible, and found the course effective in supporting rheumatoid arthritis knowledge acquisition. However, some had unmet support needs (eg, emotional, social, or interpersonal support) and desired contact with a health care provider to support course content. As in our study, participants also expressed a desire for additional support to reinforce course content. Taken together, these findings suggest that for some people, an e-learning course alone may be insufficient to address their emotional and informational needs related to their health condition. Hence, future research assessing e-learning, when delivered in combination with health professional oversight, is warranted and is currently underway [22].

While participants in our study reported various physical benefits associated with completing the course (such as reduced joint pain, increased physical abilities, and weight loss), its true value appeared to lie in enhancing psychological well-being. Participants experienced reduced fear and worry about osteoarthritis and its prognosis, accompanied by newfound optimism for the future. Notably, participants expressed relief in discovering that they could lead fulfilling lives despite having osteoarthritis without worsening the condition or hastening the need for joint replacement surgery. These findings are particularly important given the substantial psychosocial impact of living with osteoarthritis. For instance, a recent systematic review comprising 21 qualitative studies (n=665) highlighted the impact that a perceived poor prognosis (such as the belief that osteoarthritis is a “progressive degenerative disease”), the perceived necessity for surgery, and one’s preoccupation with joint damage have on the psychological well-being of people with knee osteoarthritis [23]. These beliefs negatively impacted quality of life and led to feelings of hopelessness, loss of control, grief, and inadequacy.

Despite the overall positive views regarding e-learning observed in our study, all participants described the course as time consuming and repetitive at times. This led some to “skim” various sections of content, particularly when they felt it did not align with their individual needs (eg, the body weight module). Many also wanted continuous access to revisit content rather than time-limited access. To reduce time pressure and better enable participants to learn at their own pace and more easily tailor how they engage with the course (eg, skip sections that feel repetitive or revisit sections at a later date to reinforce learning), we have since made the course available on the FutureLearn platform with free, unrestricted access. Future work should focus on developing strategies for large-scale implementation and dissemination.

### Strengths and Limitations

Several strengths and limitations should be acknowledged. We used purposive sampling to ensure a variety of participants were included. To facilitate accurate recall, all interviews were conducted within a month of completing involvement in the RCT. To enhance rigor and validity, 2 researchers (RKN and LS) independently coded the data, with 1 (LS) having no involvement in course development. All researchers contributed to theme generation using the matrix of codes and quotes, and these processes also provided opportunities for reflexive discussion and critically challenging interpretations. There were several limitations. Despite the use of purposive sampling, selection bias may be present, as participants with more favorable opinions about the e-learning course may have been more inclined to provide consent to be interviewed. We did attempt to overcome this by deliberately recruiting people with lower perceived course usefulness; however, we purposely did not recruit those who reported that they found the course “not useful” at all in the RCT. This was because the 6 participants in the RCT who reported this also reported not having accessed the course (one because they believed the course required payment and the others for unknown reasons). While their exclusion was deemed appropriate to address our research question, it may nevertheless bias findings toward more favorable interpretations, as the perspectives of those who disengaged entirely were not captured. Finally, researcher perspectives could have affected findings, as most authors were involved in the development of the e-learning course. We attempted to address this by ensuring that the interviewer (who was also one of the independent coders) had no previous involvement in either the e-learning course development or its RCT evaluation.

### Conclusions

In summary, participants in this qualitative study generally found the e-learning course beneficial, noting changes in their beliefs and attitudes toward osteoarthritis and its management. These shifts included understanding that osteoarthritis progression is not inevitable, the joint does not require constant protection, and joint replacement is not an inevitable outcome. For this sample, such changes appeared to foster a more positive mindset, instilling newfound confidence in living with osteoarthritis and a more optimistic outlook for the future. The e-learning course is now available with free, unrestricted access and may serve as a useful resource to support people with osteoarthritis in improving their understanding of the condition and its management.

## Appendix

Multimedia Appendix 1 Participant details.

Multimedia Appendix 2 Themes, subthemes, and example quotes.

## Abbreviations

COREQ: Consolidated Criteria for Reporting Qualitative Research
RCT: randomized controlled trial
REDCap: Research Electronic Data Capture

## Data Availability

The datasets generated or analyzed during this study are available from the corresponding author on reasonable request.
