# Supplementary material for: Impact of a Consumer e-Learning Course on Beliefs, Treatment Choices, and Outcomes Among People With Hip and Knee Osteoarthritis: Qualitative Interview Study
Source: JMIR Aging. 2025 Nov 5;8:e80282. doi: 10.2196/80282 (PMC12631089; doi:10.2196/80282)
Supplement: Multimedia Appendix 1 [file aging_v8i1e80282_app1.pdf]

## Multimedia Appendix 1. Participant details (N=20)

| Alias | Most painful joint | Age (y) | Sex | Level of education completed | Geographic location[a] | Symptom duration in the most painful joint (y) | Knee pain[b] (RCT[c]: 0 wk assessment) | Osteoarthritis knowledge[d] (RCT: 0 wk assessment) | Self-reported e-learning modules completed (maximum 4) | Overall perceived usefulness of e-learning[e] (RCT: 5 wk assessment) | Change in osteoarthritis knowledge[d] (RCT: 5 wk assessment | Change in osteoarthritis knowledge[d] (RCT: 13 wk assessment) |
|---|---|---|---|---|---|---|---|---|---|---|---|---|
| Adam | Knee | 62 | Male | University or tertiary institute | Rural or regional | 15 | 2 | 38 | 1, 2, 3, and 4 | Extremely | 12 | 12 |
| Patricia | Knee | 71 | Female | University or tertiary institute | Metropolitan | 9 | 6 | 41 | 1, 2, 3, and 4 | Moderately | 13 | 11 |
| Helen | Knee | 78 | Female | Secondary or high school | Metropolitan | 9 | 6 | 42 | 1,2, and 3 | Extremely | 10 | 10 |
| Rebecca | Knee | 59 | Female | Higher university degree | Rural or regional | 19 | 2 | 39 | 1,2,3, and 4 | Moderately | 16 | 16 |
| Kylie | Knee | 84 | Female | Trade or trade certificate | Metropolitan | 15 | 8 | 41 | 1 and 2 | Slightly | 5 | 3 |
| Cathryn | Knee | 73 | Female | Secondary or high school | Metropolitan | 15 | 4 | 44 | 1,2,3, and 4 | Extremely | 11 | 11 |
| Amelia | Hip | 68 | Female | Secondary or high school | Metropolitan | 2 | 6 | 37 | 1,2,3, and 4 | Moderately | 16 | 14 |
| James | Knee | 79 | Male | Trade or trade certificate | Rural or regional | 30 | 5 | 41 | 1,2,3, and 4 | Slightly | 14 | 7 |
| Grace | Hip | 72 | Female | Secondary or high school | Rural or regional | 5 | 6 | 32 | 1,2,3, and 4 | Moderately | 19 | 9 |

| | | | | | | | | | | | | |
|---|---|---|---|---|---|---|---|---|---|---|---|---|
| Liz | Knee | 79 | Female | University or tertiary institute | Rural or regional | 1 | 6 | 35 | 1,2,3, and 4 | Extremely | 18 | 5 |
| Karen | Hip | 71 | Female | University or tertiary institute | Metropolitan | 3 | 6 | 33 | 1,2,3, and 4 | Extremely | 18 | 18 |
| Tim | Knee | 75 | Male | Trade or trade certificate | Metropolitan | 0 | 5 | 31 | 1,2,3, and 4 | Extremely | 19 | 24 |
| Mary | Knee | 70 | Female | Trade or trade certificate | Metropolitan | 12 | 3 | 37 | 1,2,3, and 4 | Extremely | 4 | 7 |
| Naomi | Hip | 64 | Female | Trade or trade certificate | Rural or regional | 12 | 6 | 34 | 1,2,3, and 4 | Extremely | 8 | 17 |
| Jane | Hip | 63 | Female | Secondary or high school | Metropolitan | 1 | 6 | 37 | 1,2,3, and 4 | Extremely | 17 | 16 |
| Simon | Knee | 62 | Male | University or tertiary institute | Metropolitan | 2 | 7 | 32 | 1 and 2 | Extremely | 20 | 20 |
| Jodie | Knee | 60 | Female | Trade or trade certificate | Metropolitan | 20 | 10 | 36 | 1 | Moderately | –5 | 7 |
| Michelle | Knee | 63 | Female | University or tertiary institute | Metropolitan | 3 | 4 | 38 | 1,2,3, and 4 | Slightly | 7 | 12 |
| Samantha | Knee | 69 | Female | University or tertiary institute | Metropolitan | 2 | 2 | 32 | 1,2,3, and 4 | Moderately | 22 | 16 |
| Alex | Knee | 77 | Female | University or tertiary institute | Metropolitan | 0 | 3 | 30 | 1 and 2 | Moderately | 15 | 9 |
| Mean (SD) for continuous variables | —[f] | 70 (7.3) | —[f] | —[f] | —[f] | 8.8 (8.3) | 5.2 (8.3) | 36.5 (8.3) | —[f] | —[f] | 13.0 (6.6) | 12.2 (5.4) |

[a]Defined according to The Australian Statistical Geography Standard Remoteness Structure.

[b]Self-reported average walking knee or hip pain in the past week rated on a numeric rating scale, ranging from 0 to 10, where lower scores indicate less pain.

[c]RCT: randomized controlled trial.

[d]Measured via the Osteoarthritis Knowledge Scale, ranging from 11 to 55, where higher scores indicate more accurate knowledge.

[e]Measured via a 4-point scale with the following options: 1=not at all useful; 2=slightly useful; 3=moderately useful; 4=extremely useful.

[f]Not applicable.
