# Supplementary material for: Impact of a Consumer e-Learning Course on Beliefs, Treatment Choices, and Outcomes Among People With Hip and Knee Osteoarthritis: Qualitative Interview Study
Source: JMIR Aging. 2025 Nov 5;8:e80282. doi: 10.2196/80282 (PMC12631089; doi:10.2196/80282)
Supplement: Multimedia Appendix 2 [file aging_v8i1e80282_app2.pdf]

Multimedia Appendix 2. Themes, sub-themes, and exemplary quotes

**Theme 1: Reshaped beliefs and attitudes about OA and its management**	
Learnt progression is not inevitable	"It's not going to keep getting worse to the point where you just can't function anymore. You just keep it moving and you can work through any small pain you had and get the weight off your body so that there's less stress. But you'll basically just cope with it forever. I mean that was probably the best thing about it… it's not like a death sentence or anything" Adam<br><br>"And also, the fact that it's not going to get worse with age. That's, I think, one of the biggest things that I gained from the course…that it won't wear out more just because I get older." Jane<br><br>"That it wasn't necessarily going to progress and get worse…That I wasn't going to be in pain and crippled for the rest of my life basically." Jodie
Learnt that the joint doesn't need 'protecting'	"I'm sort of satisfied that I don't have to take it carefully because the arthritis is going to get worse, nothing's going to get worse. So yes, I'll just continue to exercise." James<br><br>"A bit was [new information] because I suppose, from the doctors that I'd spoken to, I was meant to be protecting my knee…" Cathryn<br><br>"But also, the activities. I felt that doing a lot of activities might have been doing more damage, and in the course it's, "No, go for it, do these activities, don't sit back. The more that you do, the better it is." So that's encouraging, too." Naomi
Learnt surgery is not the only option	"I guess there's two things I learnt from the course – the fact that the course said just because you've got osteoarthritis doesn't mean to say you have to go and have a joint replacement." Michelle<br><br>"I also learned that having knee replacements and hip replacements is not something that's inevitable. So that was a complete change in my information..." James<br><br>"It was extremely helpful to me. I was quite amazed really to learn that I wasn't automatically going to have to have surgery on my hip. I just assumed that that was going to happen because that's what happened with my knees. And it was really very enlightening…That was very encouraging to me. Because I just thought it was automatically going to be surgery." Karen
Reinforced the importance of recommended	"It just sort of reinforced my thoughts that there may be other ways of improving. Yeah. Dealing with it, managing it, I should say." Patricia<br><br>"I knew a lot of it, but it reinforced it in a positive way, and coming from a medical influence, it was great, ." Helen

self-management approaches	"So, for me, it was gathering more information and keeping my options open. And I suppose also supporting the decision that I'd already made; that I will keep moving and I will last longer than what the specialists say." Rebecca
**Theme 2: Adopted a proactive approach to management**	
Increased use of physical activity and exercise	"We got our bikes out over summer and things like that and trying to do a lot more hiking and things like that that we'd sort of stopped doing. Some of it because I was worried about my knees. Now I'm not worried at all about it." Adam<br><br>"So I'd barely been back [to the gym] since the surgeon has said – you know, because kind of I'd – you know, you kind of hear something like that and then you go, "Hmm, OK, how" – then if steps aren't good the Body Balance is probably not good because it's lots of standing on one leg and doing all of that. But I've gone back to it now. Which I love." Rebecca<br><br>" I've been going back to classes two or three times a week. Strength and balance classes and the pain has gone from my hip." Karen<br><br>"I do a dance class as well. And I wasn't doing that previously. So that's new." Jane
Prompted care-seeking	"There was reference to getting back with your physiotherapist and getting some exercises from the physio. I did that as well…So that in itself came out of the course." Tim<br><br>"It certainly reminded me to talk to my physio more about my knee rather than my doctor…so it was very easy for me to take that out of it." Rebecca<br><br>"And I'm seeing a physio…So I'm trying to – yes, I'm trying to do all the stuff that they had in – you know, all the information they had in that program." Helen<br><br>"Because even though the course gave me a lot of ideas of what to do non-surgically, I'm following up now – maybe I do need surgery." Jodie
**Theme 3: Now a more positive mindset**	
Less fear and worry	"The biggest thing that surprised me was just not worrying about it because it's not like a death sentence or anything…Now I'm not worried at all about it." Adam

	"And both of the orthopods that I've seen in the past 10 years have said, "Well you'd be mad to jog on that knee." And now I've started jogging again…[Before] I was worried that my knee was going to be more damaged if I put too much weight on it." Cathryn<br><br>"It's been positive. The information that I got out of the course sort of took a lot of the anxiety away…." Tim<br><br>"And look, the whole mind – my mindset changed, OK. Where I was frightened – well not frightened, but wary of doing the exercise and things like that, I followed it and did it a bit at a time. And the more I did it, it actually helped." Jodie
Increased confidence to keep living	"Now I'm kind of like, "I will be walking. I'm not going to need to have one of those little scooter things while we travel around. The caravan will be something we use for a lot longer," and that kind of stuff." Kylie<br><br>"Before you may have used it as a crutch and said, "OK, well I'm not doing that because I've got sore knees." Whereas, you know, after doing the course, it's like, well, you know, just get on and do it." Simon<br><br>"I probably would have hesitated with doing a lot of the walks [when on holidays]. And that's when we went swimming with the whale sharks, we were out three hours up the coast and out very deep in the water. I might have not done that because of worry about the pain and the hips and coping. But I thought, why not? We'll just do what we – you know, I guess it's made me more confident that I can't, I won't do more damage. It's better for me than not better for me." Mary
Positivity, hope and optimism for the future	"I'm more positive than I was before the course." Grace<br><br>"Thinking well, this is it, doom and gloom, this is what I've got to look forward to. But now it's, oh no, it's not what I've got to look forward to, I can make my life better. I won't be having to go through the surgery if I can do these things and improve my joints and give that a crack first. It is definitely uplifting." Naomi<br><br>"It's [changed] the way you approach things. You know, you approach them positively as opposed to negatively. You don't not do things. You just do things and go, "OK, well this might have some discomfort, it might not." But if you don't try, you don't know." Simon
**Theme 4: Supporting learning and shifts in beliefs**	
Information perceived as trustworthy	"For me, I was after information. I didn't need to be persuaded that it was accurate information; I'd already checked about the University of Melbourne and their research beforehand, so I'd already made my decision about the reliability of the information before I went on." Kylie

	"One more thing there, the fact that I was given this information from people who knew what they were talking about meant here I am reading something on my computer screen that is from someone who's got the authority to talk about it." James<br><br>"The information was quite solid." Amelia
Appreciation for the variety of learning formats and resources	"That was probably what I liked most about it. Was to be able to expand further on it if you wished to." Patricia<br><br>"Some of the comments were very interesting and although I can't think of some of the names of the people off-hand now, I tended to follow a few different people. People who were more positive than negative. And people who found a way around things that were hard for them." Grace<br><br>"I loved the fact that they presented information and then there was like a little quiz at the end, because that shows you what you've learned. And if you got it wrong, it gave you the right information. So I think – I liked that because it was a little bit of revision." Samantha
Repetition & time investment	"I guess you could skim over the parts that you felt confident with, or that you felt were repetitive. Which I did…So not – that's probably my only negative; that it was repetitive." Jane<br><br>"Well, you get a page, and it was really making a point, and that point might be that losing weight, well it improved the arthritis. But instead of just saying that sentence, it gave a great big, long page full of words and maybe even interviews and things as well, which said exactly the same thing." James<br><br>"I felt that was a little bit repetitious. It went on – like I thought, "Yes, I've got that," and then there was more on that. And then, yes, there was – like I said, there were a lot of links to go to, but I don't know if I'm normal or not but I'm less inclined to be going out to external links." Samantha
Additional assistance desired	"I did like – I'll just tell you quickly another thing I thought that the moderators, whoever's running that course should have had more real-time input or close to real-time input into the commentary on the thread. I thought that was really lacking… it would've felt more personal to me if the moderators of the course had been more active on the threads." Amelia<br><br>"Well, someone like you who, you know, "How you going? Where you up to? Do you need any help?" Just some general advice. I mean people my age – I mean we're better with technology, I guess, than we used to be. But I'm still a person that likes to talk on the phone rather than text, and things like that." Jodie
